# Evaluation of the Effect of Patient Education and Strengthening Exercise Therapy Using a Mobile Messaging App on Work Productivity in Japanese Patients With Chronic Low Back Pain: Open-Label, Randomized, Parallel-Group Trial

**DOI:** 10.2196/35867

**Published:** 2022-05-16

**Authors:** Naohiro Itoh, Hirokazu Mishima, Yuki Yoshida, Manami Yoshida, Hiroyuki Oka, Ko Matsudaira

**Affiliations:** 1 Medical Affairs Department Shionogi & Co, Ltd Osaka Japan; 2 Data Science Department Shionogi & Co, Ltd Osaka Japan; 3 Department of Medical Research and Management for Musculoskeletal Pain, 22nd Century Medical and Research Center Faculty of Medicine The University of Tokyo Tokyo Japan

**Keywords:** mobile app, patient education, chronic low back pain, exercise regimen, mobile phone

## Abstract

**Background:**

Artificial intelligence–assisted interactive health promotion systems are useful tools for the management of musculoskeletal conditions.

**Objective:**

This study aimed to explore the effects of web-based video patient education and strengthening exercise therapy, using a mobile messaging app, on work productivity and pain in patients with chronic low back pain (CLBP) receiving pharmacological treatment.

**Methods:**

Patients with CLBP were randomly allocated to either the exercise group, who received education and exercise therapy using a mobile messaging app, or the conventional group. For patient education, a web-based video program was used to provide evidence-based thinking regarding the importance of a cognitive behavioral approach for CLBP. The exercise therapy was developed in accordance with the recommendations for alignment, core muscles, and endogenous activation, including *improvement of posture and mobility for proper alignment*, *stimulation and/or strengthening of deep muscles for spinal stability*, and *operation of intrinsic pain* for the *activation of endogenous substances by aerobic exercise*. Both groups continued to receive the usual medical care with pharmacological treatment. The end points were changes in work productivity, pain intensity, quality of life, fear of movement, and depression. The observation period for this study was 12 weeks. An analysis adjusted for baseline values, age at the time of consent acquisition, sex, and willingness to strengthen the exercise therapy was performed.

**Results:**

The exercise and conventional groups included 48 and 51 patients, with a mean age of 47.9 years (SD 10.2 years; n=27, 56.3% male patients) and 46.9 years (SD 12.3 years; n=28, 54.9% male patients) in the full analysis set, respectively. No significant impact of these interventions on work productivity was observed in the exercise group compared with the conventional group (primary end point: Quantity and Quality method; 0.062 vs 0.114; difference between groups −0.053, 95% CI −0.184 to 0.079; *P*=.43). However, the exercise group showed consistently better trends for the other end points than did the conventional group. Compared with the conventional group, the exercise group showed a significant improvement in the symptoms of low back pain (3.2 vs 3.8; difference between groups −0.5, 95% CI −1.1 to 0.0; *P*=.04), quality of life (EuroQoL 5 Dimensions 5 Level: 0.068 vs 0.006; difference between groups 0.061, 95% CI 0.008 to 0.114; *P*=.03), and fear of movement at week 12 (−2.3 vs 0.5; difference between groups −2.8, 95% CI −5.5 to −0.1; *P*=.04).

**Conclusions:**

This study suggests that patient education and strengthening exercise therapy using a mobile messaging app may be useful for treating CLBP. This study does not reveal the effect of therapeutic interventions on CLBP on work productivity. Thus, further research is required to assess work productivity with therapeutic interventions.

**Trial Registration:**

University Hospital Medical Information Network Clinical Trials Registry UMIN000041037; https://center6.umin.ac.jp/cgi-open-bin/ctr_e/ctr_view.cgi?recptno=R000046866

## Introduction

### Background

Chronic low back pain (CLBP) is common in adults, with prevalence rates as high as >80% [1,2]. In Japan, the low back is the most common site for pain in 31% of Japanese adults aged ≥20 years [3].

Low back pain (LBP) is associated with high disability. In the Global Burden of Diseases, Injuries, and Risk Factors Study 2017, LBP ranked highest in terms of years lived with disability among the 354 conditions studied over the period of 28 years [4]. Recurrence of pain, limitation of activity, loss of productivity, and work absenteeism contribute to the associated huge socioeconomic burden of CLBP [5-7].

In a retrospective, cross-sectional study using the 2014 Japan National Health and Wellness Survey data, 77.4% of 30,000 Japanese adults with CLBP reported presenteeism and had a poor quality of life (QoL) compared with those without presenteeism [8]. A cross-sectional survey of 392 patients with CLBP in Japan estimated the costs for lost productivity as approximately ¥1.2 trillion (US $10 billion) per year [7]. A recent internet-based survey of 10,000 Japanese workers reported that 36.8% of the participants had a health problem that interfered with their work during the past 4 weeks. Among the symptoms that most affect presentism, neck pain or shoulder stiffness, LBP, and mental illnesses accounted for approximately 35.7%. The annualized costs of presenteeism per capita for these conditions were US $414.05, US $407.59, and US $469.67, respectively [9].

Several studies have reported that exercise alleviates CLBP and disability [10-12]. Furthermore, exercise regimens have been reported to reduce disability [13] and improve the QoL of individuals with CLBP [14,15]. Patients with chronic pain, including CLBP, exhibit various symptoms and signs as the duration of the pain increases. When the pain lingers, it becomes intractable and serious through a cyclical interaction with psychosocial factors. As illustrated by the fear-avoidance model of pain, pain often involves catastrophizing when it becomes intractable [16]. There are also several psychological treatments or therapies for musculoskeletal symptoms [17]. In a study on patients with CLBP, both groups—one that received only exercise therapy and the other that received a combination of cognitive behavioral therapy and exercise therapy—showed improvements in pain intensity and QoL compared with baseline [18].

Despite these encouraging results, patients often show noncompliance with exercise therapy. Perceptions of the underlying illness and exercise therapy, lack of positive feedback, and degree of helplessness are factors related to noncompliance with exercise therapy [19]. In recent years, digital devices have become popular for supporting exercise therapy for musculoskeletal pain [20-22]. These digital devices have been reported to improve adherence [23,24]. Most studies have supported the role of digital interventions for LBP alleviation [24-27].

The mobile messaging app Secaide (Travoss Co, Ltd) is a digital device designed to enhance the patient’s understanding of CLBP and enable remote exercise therapy for more accessible and personalized home-based pain management. The app was nicknamed *se · ca · ide* by the *self-care guide service*. *Secaide* also means *in the world* when read in Japanese. The usefulness of mobile messaging app–based interventions in managing neck and/or shoulder stiffness and LBP is established in workers in randomized controlled trials [28].

### Objectives

Previous studies have not clarified the impact of intervention in CLBP treatment on presenteeism in patients. As a hypothesis, we expected that therapeutic intervention for CLBP would have a positive effect on presenteeism. This study aims to explore the effects of patient education and strengthening exercise therapy on work productivity, symptoms, and QoL in patients with CLBP who were receiving medication and who continued to experience pain despite treatment. In a new attempt, we used web-based videos for patient education and a mobile messaging app to support the continuation of exercise therapy. Because of the COVID-19 pandemic, we devised methods for study continuation without any visits to clinics by the intervention in web-based remote exercise therapy and by using patient-reported outcomes (PROs) as an outcome evaluation method.

## Methods

### Study Design

**Figure 1:**
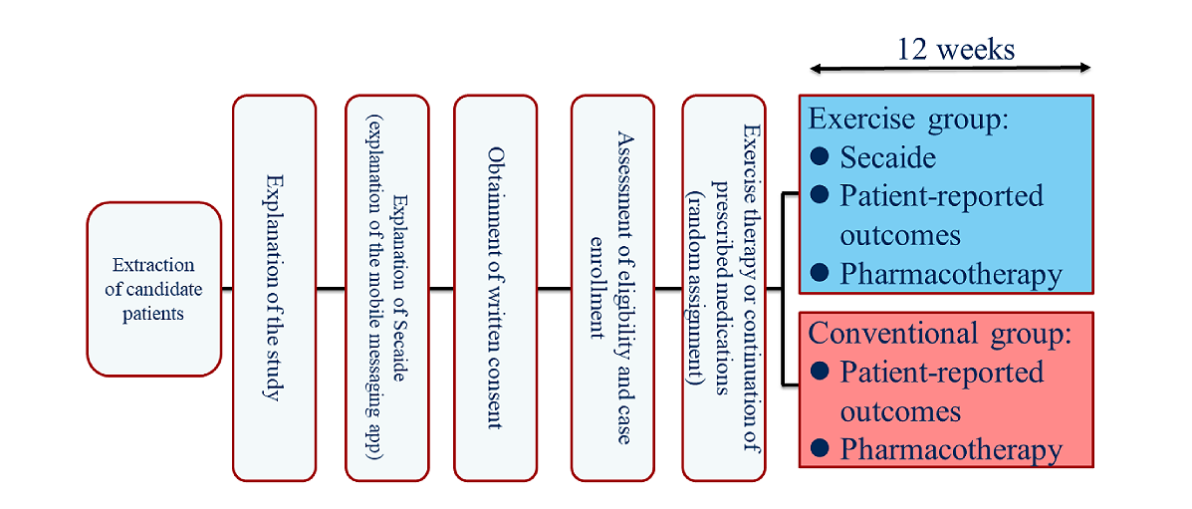
Study design.

This was a multicenter, open-label, randomized, parallel-group study conducted in Japan from June 2020 to March 2021 at 16 clinics (Multimedia Appendix 1). The main clinical specialty of the 16 community-based clinics included 8 (50%) orthopedic facilities, 3 (19%) pain clinics, and 5 (31%) primary care facilities. In this study, patients were followed up for 12 weeks (Figure 1). Patients who met the eligibility criteria were randomly assigned using a stochastic minimization procedure with allocation regulators, such as age (<45 or ≥45 years), sex (male or female), and willingness to enhance exercise therapy (yes or no).

### Ethics Approval

The study was conducted in accordance with all the international and local laws, the principles of the *Declaration of Helsinki*, and the SPIRIT (Standard Protocol Items: Recommendations for Interventional Trials) statement [29]. Written informed consent was obtained from all patients before enrollment in the study. The study protocol and all subsequent amendments were approved by the institutional review board of Takahashi Clinic (clinical research implementation plan MA2020-P-002). The study was registered with the University Hospital Medical Information Network Clinical Trials Registry (UMIN000041037).

### Study Population

Patients who met the following criteria were included in the study: (1) having LBP for >3 months, (2) aged 20 to 64 years, (3) receiving prescribed pharmacological treatment for the pain, (4) not likely to experience any unexpected pain flare-ups for 12 weeks, (5) able to walk independently, (6) engaging in work for >3 days per week in either full-time or part-time capacity for >3 hours a day, and (7) having the skill and understanding to operate mobile communications. The CLBP diagnosis was established by qualified practicing physicians.

The key exclusion criteria were as follows: (1) aged >65 years, (2) having CLBP unrelated to a musculoskeletal condition, (3) with radiculopathy or constructive spinal deformity, (4) having LBP with red flags (with chest pain, malignant tumor, HIV infection, malnutrition, significant weight loss of ≥5% within 1 month, extensive neurological symptoms, or fever of ≥37.5 °C), (5) using over-the-counter medications for CLBP, (6) pregnant women and those who were willing to be pregnant during the clinical trial period, (7) receiving steroids (intravenous injection or oral administration) or opioids, and (8) unable to understand the Japanese language.

### Study Treatment, Education, and Therapy

The patients received the prescribed pharmacological treatment, surgical treatment, and/or patient education and exercise therapy for the management of CLBP.

#### Pharmacological Treatment

Information about the use of medications for pain was obtained from an electronic medical record system (Mebix, Inc). Pharmacological treatment included nonsteroidal anti-inflammatory drugs, acetaminophen, weak opioids, blood flow improvers, muscle relaxants, medications for osteoporosis, antidepressant drugs, steroids, antiepileptic drugs, and nerve-blocking agents, such as local anesthetic drugs. Medications were assessed at randomization; weeks 4, 8, and 12; and study discontinuation.

#### Surgical Treatment

Any surgeries for pain relief were recorded at randomization; weeks 4, 8, and 12; and study discontinuation.

#### Patient Education and Exercise Therapy

A web-based video program was used to provide evidence-based thinking regarding the importance of a cognitive behavioral approach for patients with CLBP. The exercise therapy was developed by Travoss Co, Ltd, in accordance with the recommendations for alignment, core muscles, and endogenous activation, including *improvement of posture and mobility for proper alignment*, *stimulation and/or strengthening of deep muscles for spinal stability*, and *operation of intrinsic pain* for the *activation of endogenous substances by aerobic exercise* [30,31].

**Figure 2:**
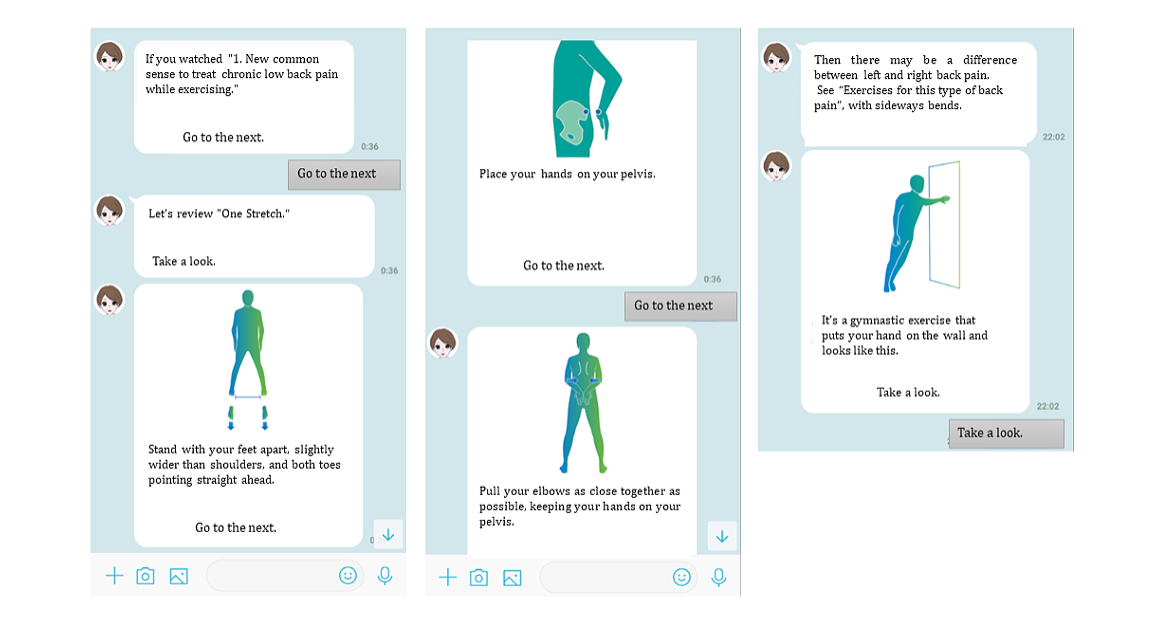
Examples of exercises with instructions from the artificial intelligence–assisted health program (Secaide).

**Figure 3:**
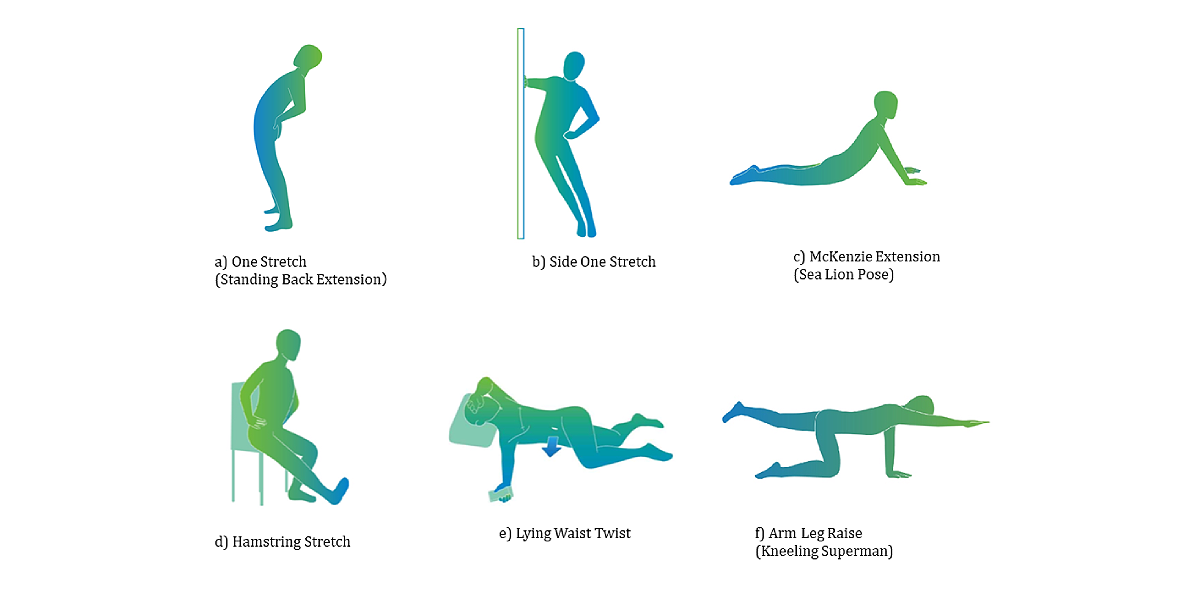
Exercise menu on Secaide.

**Figure 4:**
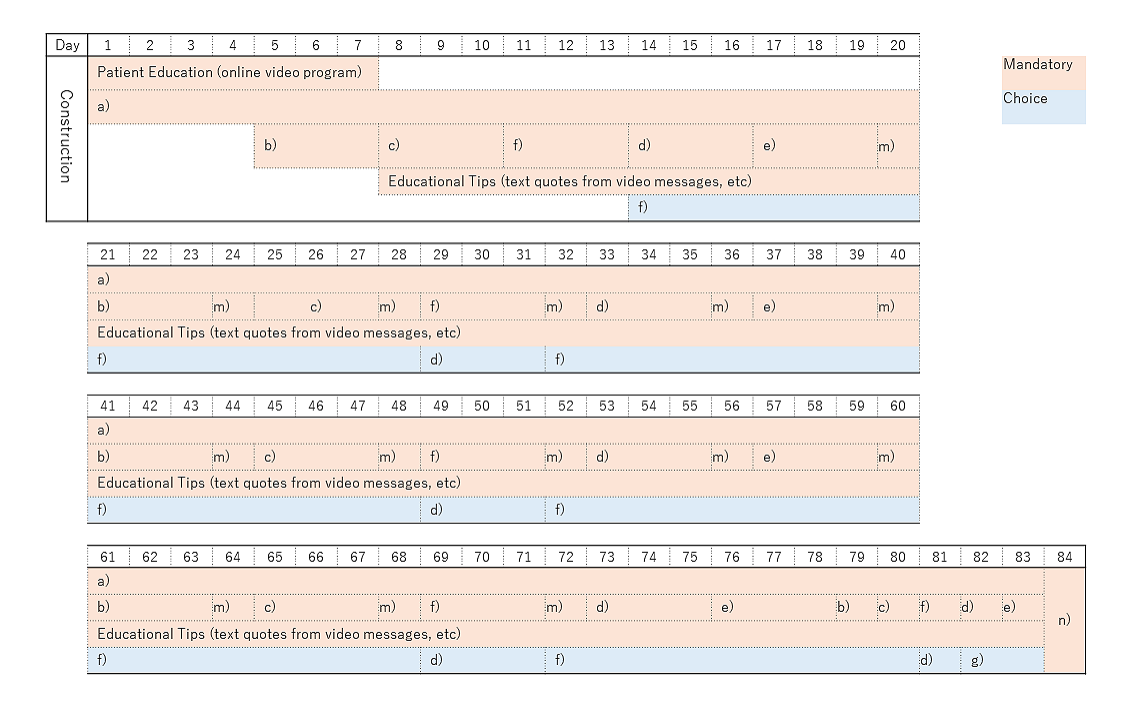
Exercise schedule on Secaide. a) One Stretch (Standing Back Extension), b) Side One Stretch, c) McKenzie Extension (Sea Lion Pose), d) Hamstring Stretch, e) Lying Waist Twist, f) Arm Leg Raise (Kneeling Superman), m) Mindfulness, n) Questionnaire.

Secaide, a mobile messaging app for mobile communication devices such as smartphones and tablets, with download enabled by a QR code, is an aid to exercise therapy. In Japan, this mobile messaging app is used for SMS text messaging and voice calls [28]. Patient education and exercise therapy announcements were conducted as follows. The artificial intelligence–assisted chatbot was programmed to send messages to users with exercise instructions and some tips on what they can do in their daily lives to improve their symptoms. The messages were sent every day at a fixed time through the LINE app (a smartphone app widely used for sending and receiving SMS text messages, images, and videos, and making voice calls in Japan; LINE Corporation). The notification time can be changed by users to a time convenient for them. The exercise was performed during the patient’s favorite time. The participants can complete their exercise within approximately 1 to 3 minutes each day (Figures 2-4). During the first week, Secaide provided evidence-based thinking about the importance of a cognitive-behavioral approach for CLBP to patient education. Secaide also provided guidance to carry out six simple exercise menus for 60 days. After the 14th, information on two types of exercise was optionally added to patients who desire further exercise. At each clinic, the conventional group received only routine medical care. In the exercise therapy group, in addition to the routine medical care, patient education and strengthening of exercise were provided. To avoid cross-contamination between the 2 groups, only the exercise group received patient education and daily exercise therapy via Secaide (Figures 2-4).

### Survey

All patients were required to respond to a web-based survey that captured demographic and background information, including occupation and exercise habits. Furthermore, pharmacological and surgical treatment for CLBP and the number of institutional visits in the last 30 days were collected at weeks 0 to 4, weeks 4 to 8, and weeks 8 to 12 and at study discontinuation.

Adherence to the use of mobile messaging app–based exercise therapy was measured by the rate of implementation (%), calculated as follows: (access days/observation period)×100. Category aggregation for the adherence rate was performed by 0% to 25%, by 25% to 50%, by 50% to 75%, and by ≥75%. Assessments were made from the log information (date) of Secaide and the PRO response date, that is, weeks 0 to 4, weeks 4 to 8, weeks 8 to 12, and weeks 0 to 12.

### Study End Points

#### Primary End Point

The primary end point was the change in work productivity at week 12. The work productivity was measured using the Quantity and Quality method (QQ method), which evaluates work productivity in terms of quality, quantity, and efficiency and is an evaluation index for absenteeism [32].

#### Secondary End Points

The secondary end points were changes in work productivity measured using the Work Productivity and Activity Impairment Questionnaire: General Health (WPAI-GH) [33], CLBP and shoulder stiffness (Numerical Rating Scale [NRS]) [34], subjective ratings of stiffness and LBP on a scale of 1 to 5 [28], disease-specific QoL (Roland-Morris Disability Questionnaire [RDQ-24]) [35,36], health-related QoL (EuroQoL 5 Dimensions 5 Level [EQ-5D-5L]) [37,38], fear of movement (Tampa Scale for Kinesiophobia [TSK-11]) [39,40], degree of depression (Kessler Screening Scale for Psychological Distress [K-6]) [41], drug use, and consultation status at medical institutions. All the secondary end points were measured at baseline and week 12. In addition, changes in LBP and drug use were measured at weeks 4 and 8 during the study period.

### Statistical Analysis

The data related to changes in WPAI-GH in a 6-week randomized study of patients with LBP were used to calculate the sample size of 100 participants [42]. The required sample size in this study was estimated to be 90 patients for 80% power at an intergroup difference of 2.7, a common SD of the 2 groups of 4.5, and an α level of .05, using the 2-sample, 2-tailed *t* test. Considering a dropout rate of 10%, the total sample size was 100 (n=50, 50% patients in each group). For allocation, a minimization method was used, with adjustments for age, sex, and willingness to adopt the exercise therapy.

Data were summarized using descriptive statistics of the mean (SE) for continuous variables and frequencies and percentages for categorical variables. To compare continuous data in the 2 groups, an analysis of covariance model (covariates: treatment, baseline, age, sex, and willingness to adopt the exercise therapy) or mixed-effects model for repeated measures (covariates: treatment, baseline, time, time×treatment, age, sex, and willingness to adopt the exercise therapy) was used for the primary and secondary end points, depending on the times of measurements. The Fisher exact test was used to compare the percentages in the 2 groups.

In patients who had data reported at week 12, post hoc analyses were performed to check the impact of the treatment compliance (<75% and ≥75% exercise groups and conventional group) on the primary end point (work productivity) and secondary end points (NRS of CLBP and RDQ-24). Data were analyzed using SAS (version 9.4; SAS Institute Inc).

## Results

### Study Population

**Figure 5:**
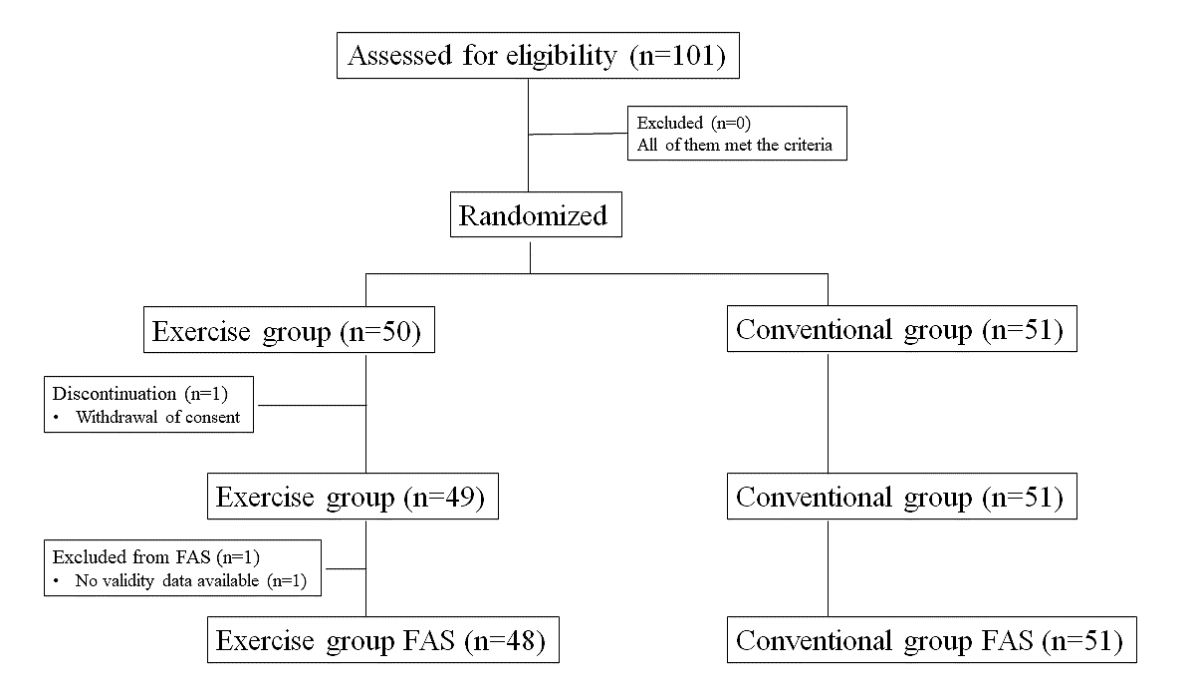
Patient disposition. FAS: full analysis set.

A total of 101 patients with CLBP were recruited, and consenting participants were randomly allocated to either the exercise group (n=50, 49.5% randomized; n=48, 47.5% analyzed for efficacy), who used the web-based videos and Secaide for exercise therapy, or the conventional group (n=51, 50.5% randomized and analyzed; Figure 5). Both groups continued with the prescribed pharmacological treatments.

**Figure 6:**
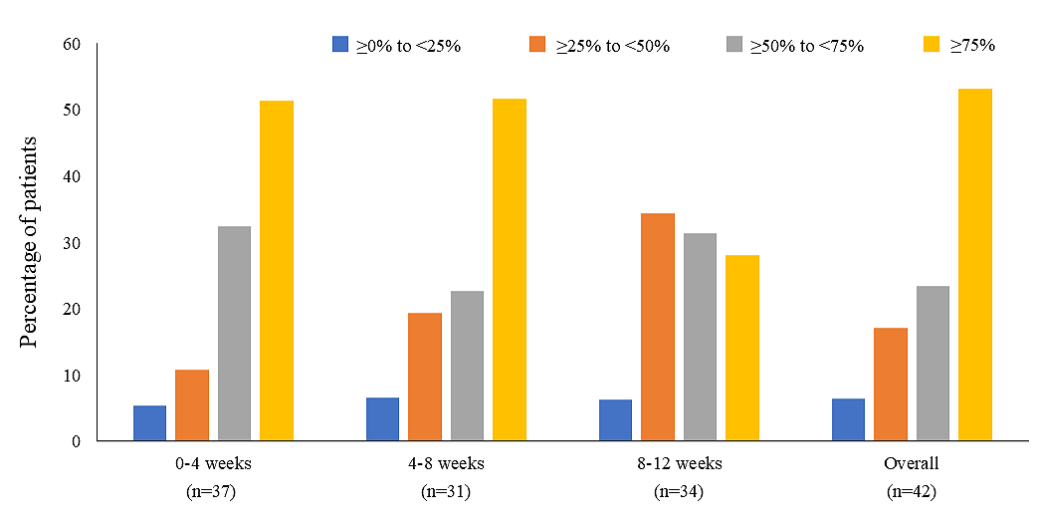
Compliance rates for the use of mobile messaging app–based exercise therapy during the study duration. Exercise status is evaluated by access log to Secaide within a specified period. Percentage of patients (%)=(access days/observation period)×100. Category aggregation for the rate of adherence was performed by 0% to 25% (blue), 25% to 50% (orange), 50% to 75% (gray), and ≥75% (yellow).

The baseline characteristics of patients in the exercise and conventional groups are shown in Table 1. No difference in many characteristics was observed between the 2 groups. However, variability in work productivity was observed (WPAI-GH). In addition, >85% of the patients in both groups requested exercise therapy (exercise group: 42/48, 88% patients; conventional group: 45/51, 88% patients), which was a group highly conscious of exercise. Of the 48 participants in the exercise group, 37 (77%) were adherent to the use of mobile messaging app–based exercise therapy in weeks 0 to 4, 31 (65%) in weeks 4 to 8, and 32 (67%) in weeks 8 to 12 (Figure 6).

**Table 1:** Baseline characteristics (full analysis set).

			Exercise group (n=48)		Conventional group (n=51)
**Age (years), mean (SD)**			47.9 (10.2)		46.9 (12.3)
	<45	18 (37.5)		20 (39.2)	
	≥45	30 (62.5)		31 (60.8)	
**Sex, n (%)**					
	Women	21 (44)		23 (45)	
	Men	27 (56)		28 (55)	
BMI (kg/m^2^), mean (SD)			24.42 (4.05)		23.39 (4.18)
**Duration of CLBP^a^ (years), n (%)**					
	<0.5	3 (6)		5 (10)	
	0.5 to <1	3 (6)		6 (12)	
	≥1	42 (88)		40 (78)	
**Exercise habits, n (%)**					
	Yes	14 (29)		19 (37)	
	No	14 (29)		19 (37)	
	Sometimes	20 (42)		13 (25)	
**Hope for exercise therapy, n (%)**					
	Yes	42 (88)		45 (88)	
	No	6 (13)		6 (12)	
**Work engagement, n (%)**					
	Full time (>40 hours per week)	34 (71)		40 (78)	
	Part time	14 (29)		11 (22)	
**Family structure, n (%)**					
	Living alone	10 (21)		9 (18)	
	Living with children only	1 (2)		4 (8)	
	Living with adults only	18 (38)		18 (35)	
	Living with adults and children	19 (40)		20 (39)	
**Income (¥ [US $]), n (%)**					
	<3 million (24,000)	15 (31)		10 (20)	
	3 million to <5 million (24,000 to 40,000)	14 (29)		16 (31)	
	5 million to <8 million (40,000 to 64,000)	9 (19)		13 (25)	
	≥8 million (64,000)	8 (17)		7 (14)	
	Decline to answer	2 (4)		5 (10)	
Education level (completed university education), mean (SD)			25 (52.1)		22 (43.1)
**Drink alcohol, n (%)**					
	Yes	17 (35)		18 (35)	
	No	12 (25)		22 (43)	
	Sometimes	19 (40)		11 (22)	
**Smoking, n (%)**					
	Never smoked	23 (48)		26 (51)	
	Former smoker	14 (29)		15 (29)	
	Current smoker	11 (23)		10 (20)	
**Work productivity, QQ method,^b^ mean (SD)**					
	Performance degradation	0.51 (0.303)		0.516 (0.314)	
	Days of work loss due to poor performance	10.466 (8.485)		12.409 (9.956)	
**Work productivity (WPAI-GH^c^), mean (SD)**					
	Work time	4.3 (12.4)		8.2 (21.8)	
	Impairment while working	35.3 (29.8)		45.6 (33.2)	
	Overall work impairment	37.0 (30.7)		47.7 (34.4)	
	Activity impairment	47.2 (31.6)		50.4 (29)	
**NRS,^d^ mean (SD)**					
	CLBP	5 (2.4)		5.1 (2.1)	
	Shoulder stiffness	4.5 (3.0)		4.5 (2.8)	
RDQ-24,^e^ mean (SD)			8.6 (5.3)		7.4 (4.7)
EQ-5D-5L,^f^ mean (SD)			0.720 (0.195)		0.746 (0.142)
TSK-11,^g^ mean (SD)			26.4 (6.1)		24.6 (6.6)
K-6,^h^ mean (SD)			6.2 (5.6)		5 (4.9)
**Medical institution consultation status (in the last 30 days), mean (SD)**					
	Hospital	1.9 (1.7)		2.1 (2.3)	
	Clinic	0.8 (1.6)		1.1 (2.5)	
	Acupuncture and moxibustion clinic	0.2 (0.8)		0.1 (0.2)	
	Manipulative clinic	0.8 (1.7)		0.8 (1.9)	
	Others	0.3 (1.0)		0.4 (0.9)	

^a^CLBP: chronic low back pain.

^b^QQ method: Quantity and Quality method.

^c^WPAI-GH: Work Productivity and Activity Impairment Questionnaire: General Health.

^d^NRS: Numerical Rating Scale.

^e^RDQ-24: Roland-Morris Disability Questionnaire.

^f^EQ-5D-5L: EuroQoL 5 Dimensions 5 Level.

^g^TSK-11: Tampa Scale for Kinesiophobia.

^h^K-6: Kessler Screening Scale for Psychological Distress.

### Primary End Point

At week 12, the mean change (SE) in work productivity (QQ method) in the exercise group (n=37) and the conventional group (n=32) was 0.062 (0.069) and 0.114 (0.069), respectively (difference between groups −0.053, 95% CI −0.184 to 0.079; *P*=.43). No significant difference was observed at the primary end point.

### Secondary End Points

#### Work Productivity

Changes in the WPAI-GH parameters in the 2 groups at week 12 are shown in Table 2. Percent overall work impairment due to health in the exercise group (n=36) and the conventional group (n=26) was −13.3 (SE 6.8) and −4.7 (SE 7.6), respectively (difference between groups −8.6, 95% CI −23.6 to 6.5; *P*=.26).

**Table 2:** Changes in Work Productivity and Activity Impairment Questionnaire: General Health parameters and QoL^a^ at week 12.

Parameter		Exercise group, least squares mean (SE)	Conventional group, least squares mean (SE)	Difference between groups in the 12 weeks, least squares mean (95% CI)	*P* value
**Work Productivity, n (%)**		36^b^ (100)	26 (100)	N/A^c^	
	Work time	3.8 (3.4)	1.2 (4.1)	2.7 (−5.4 to 10.7)	.51
	Impairment while working	−16.5 (6.2)	−6.8 (6.9)	−9.6 (−23.3 to 4.1)	.17
	Overall work impairment	−13.3 (6.8)	−4.7 (7.6)	−8.6 (−23.6 to 6.5)	.26
	Activity impairment	−16.7 (5.7)	−6.4 (6.7)	−10.3 (−23.6 to 3.0)	.13
**QoL scale, n (%)**		38 (100)	34 (100)	N/A	
	RDQ-24^d^	−2.1 (0.8)	−0.3 (0.9)	−1.9 (−3.7 to 0.0)	.05
	EQ-5D-5L^e^	0.068 (0.024)	0.006 (0.026)	0.061 (0.008 to 0.114)	.03

^a^QoL: quality of life.

^b^Data for activity impairment due to health were analyzed for 37 patients.

^c^N/A: not applicable.

^d^RDQ-24: Roland-Morris Disability Questionnaire.

^e^EQ-5D-5L: EuroQoL 5 Dimensions 5 Level.

#### Low Back Pain

At week 12, although no statistically significant difference in the reduction of the NRS scores was observed between the exercise (mean −1.1, SE 0.3) and conventional groups (mean −0.7, SE 0.4; *P*=.26), the mean subjective improvement in CLBP symptoms was significantly greater in the exercise group (mean 3.2, SE 0.2) than in the conventional group (mean 3.8, SE 0.3; difference between groups −0.5, 95% CI −1.1 to 0.0; *P*=.04).

#### Quality of Life

At week 12, no statistically significant differences in the RDQ-24 scores were observed between the exercise and conventional groups. A significant improvement in EQ-5D-5L at week 12 was observed in the exercise group compared with that in the conventional group (Table 2).

#### Kinesiophobia

At week 12, a significant improvement in the TSK-11 score was observed in the exercise group (mean −2.3, SE 1.2) compared with that in the conventional group (mean 0.5, SE 1.3; difference between groups −2.8, 95% CI −5.5 to −0.1; *P*=.04).

#### Depression

At week 12, no significant improvement in the K-6 score was observed in the exercise group (mean −1.5, SE 0.8) compared with that in the conventional group (mean −0.6, SE 0.9; difference between groups −0.9; 95% CI −2.7 to 0.9; *P*=.34).

#### Change in Consultation Status

Visits to clinics were significantly reduced in the exercise group at weeks 4, 8, and 12. Similarly, a significant reduction in visits to the acupuncture and moxibustion clinics was observed in the exercise group at weeks 4 and 8 (Multimedia Appendix 2).

#### Surgical Treatment and Change in Drug Use

No differences for surgical treatment or changes in drug use were observed in the conventional or exercise group throughout the study period.

#### Post Hoc Analysis

In this study, no significant difference in work productivity (QQ method), pain intensity, and RDQ-24 was observed in the exercise group. As a post hoc analysis, the effects of exercise therapy on work productivity (QQ method), pain intensity, and RDQ-24 were examined in the group with a high compliance rate of exercise (≥75%) and the other groups (<75% compliance). At week 12, patients who showed a higher (≥75%) adherence to the exercise regimen had a greater improvement in work productivity (QQ method), NRS scores, and RDQ-24 than those with <75% adherence or the conventional group (Table 3).

**Table 3:** Change from baseline of work productivity, CLBP,^a^ and quality of life among treatment compliances at week 12 (post hoc analysis).^b^

Parameters	Exercise group compliance ≥75% (n=18), least squares mean (95% CI)	Exercise group compliance <75% (n=20), least squares mean (95% CI)	Conventional group (n=34), least squares mean (95% CI)
Work productivity (QQ method^c^)	0.00 (−0.14 to 0.15)	0.05 (−0.11 to 0.21)	0.08 (−0.03 to 0.18)
CLBP (NRS^d^)	−2.28 (−3.47 to −1.09)	−0.15 (−1.03 to 0.73)	−0.91 (−1.48 to −0.34)
Quality of life (RDQ-24^e^)	−3.06 (−4.45 to −1.66)	−2.20 (−4.51 to 0.11)	−0.76 (−2.15 to 0.62)

^a^CLBP: chronic low back pain.

^b^No statistical tests were performed.

^c^QQ method: Quantity and Quality method.

^d^NRS: Numerical Rating Scale.

^e^RDQ-24: Roland-Morris Disability Questionnaire.

## Discussion

### Principal Findings

The exercise intervention is considered an integral part of CLBP management and has been reported to reduce pain and improve function in patients with CLBP; however, there are challenges in exploring effective exercise types and continuing exercise [43,44]. In recent years, various digital interventions have attempted to address these challenges [45-49].

The web-based video patient education and strengthening exercise therapy using the mobile messaging app did not show any significant changes in work productivity or loss of workdays due to CLBP at week 12 compared with the conventional pharmacological treatment in this study. To the best of our knowledge, there is no randomized controlled trial with the intervention outcome to improve work productivity in patients with CLBP; therefore, this result cannot be compared with previous studies. It is possible that drastic changes in the working environment during the COVID-19 pandemic affected the assessment of work productivity. During the research period, the Government of Japan began to recommend remote work as a national policy. In the evaluation of work productivity, the quantity and quality of work at the time of evaluation were compared with those in the absence of CLBP. The effect of changes in working style might be greater than that of exercise therapy on work productivity. A survey of workers in remote work before and during the COVID-19 pandemic conducted in Japan in 2020 also reported that full remote work of 5 days a week reduced work productivity [50]. Therefore, the difference in work productivity between the 2 groups due to exercise therapy may not have been observed. In fact, many secondary end points showed a significant improvement in exercise therapy. However, the work productivities did not show a significant improvement. The work productivity assessments may have been particularly susceptible to COVID-19 compared with outcomes such as pain intensity and QoL. To assess the impact of exercise therapy on work productivity in patients with CLBP, further improved clinical studies will be considered.

The use of mobile devices can enhance patient engagement in self-management of CLBP and improve exercise compliance [51]. In this study, >50% (36/47) of the participants had ≥75% compliance with the use of the mobile messaging app–based exercise therapy. In previous studies, similar adherence rates of about 50% to 70% for home-based exercise programs have been reported [52,53]. The results of this study also showed high adherence to the continuation of exercise therapy using mobile devices. A problem with exercise therapy is the low level of adherence to the prescribed exercises. Two systematic reviews have reported that up to 70% of participants did not adhere to the prescribed exercises [54,55]. It has been suggested that using digital devices may improve the patient’s noncompliance with exercise therapy, which is considered to have the highest level of evidence for CLBP.

In this study, many end points, rather than the primary end point, showed results similar to those of previous studies. In particular, the degree of the subjective score of pain was significantly improved in workers who received exercise therapy, which is consistent with a previous study using Secaide [28]. The end point of QoL (EQ-5D-5L) showed a significant improvement, as in previous studies using digital interventions [47,56].

Kinesiophobia is a therapeutic target with exercise regimens in the management of CLBP [57-59]. To the best of our knowledge, no study has evaluated the impact of mobile-based apps on pain-related fear in patients with CLBP. In this study, we evaluated kinesiophobia using the TSK-11 scale, which has been validated for use in patients with CLBP [60]. At week 12, a significant improvement in the TSK-11 score was observed in the exercise group. From the above results, it is considered that the effect of exercise therapy was supported in this study, as well as in previous studies.

In addition, a post hoc analysis was used to evaluate the relationship between exercise therapy adherence and outcomes. High adherence showed good outcomes in work productivity (QQ method), CLBP score (NRS), and RDQ-24 score. Recently, evaluation using PROs has attracted attention in clinical trials [61]. The concept of minimal clinically significant difference (MCID) is established, and its importance is recognized. MCID is not a statistically significant difference, but it is an indicator of the clinical benefits to patients. The MCID has been reported as an NRS ≥2 for LBP [62] and a 30% change in score for RDQ-24 (if the score is <7) [63]. In the post hoc analysis, patients with high adherence to exercise therapy showed an improvement of 2.28 in NRS in CLBP as a change from baseline and an improvement of approximately 38% in RDQ-24. These scores achieved MCID. This improvement was clinically meaningful. Previous studies have reported that apps improve exercise therapy adherence; therefore, Secaide used in this study may also play an important role in achieving better outcomes.

In this study, we adopted the Secaide app [28], an interactive health promotion system, to aid education and exercise therapy in patients with CLBP. Furthermore, adopting web-based education and mobile messaging app–based exercise therapy may reduce the number of facility visits, ensure safety, and ensure continued patient care. Pain treatment based on traditional visits in clinics may be difficult because of the COVID-19 pandemic. PROs are becoming increasingly important, and the need for remote medical care, such as digital health programs, is increasing. The use of technology can be advantageous, enabling the remote collection of data during such unprecedented times. Using digital devices, the enhancement of exercise therapy yielded better results in more end points than in routine clinical practice. These results and compliance rates are due to research conditions. Although the impact of these on treatment cannot be evaluated correctly, it is hoped that they will provide an opportunity to consider the usefulness of remote medical care in CLBP.

### Limitations

This study had certain limitations. Changes in work quality and quantity were used as outcomes for work productivity. This study was conducted during the COVID-19 pandemic, when the social working environment has evolved with the adoption of remote working. Furthermore, these changes in the work environment may have influenced the evaluation of work productivity. The study design has the inherent limitations of a short duration (12 weeks) and a small sample size (50 in each group). There have been no previous studies with the same patient population and end point, and the required number of cases was calculated using the results of secondary end point of this study. As a result, the statistical power of this study may be lower than expected. We did not assess the rate of adherence to prescribed medications, which could possibly impact work productivity outcomes with exercise therapy using the mobile messaging app. The data for the study outcomes were self-reported, and a response bias could have led to varying estimates of the severity of CLBP. Comparison of the high adherence group with the other groups should be interpreted in a limited manner because of the results of the post hoc analysis.

### Conclusions

Web-based patient education and strengthening exercise therapy using the Secaide app may be useful for enhancing the effectiveness of exercise therapy in the treatment of CLBP. In this exploratory study, the exercise group showed consistently better trends for most end points than did the conventional group. The adherence to exercise therapy improved work productivity, NRS for CLBP, and RDQ-24, suggesting that the mobile messaging app is useful for CLBP treatment.

This study did not reveal the effect of therapeutic interventions on CLBP on work productivity. Further research is required to assess work productivity with therapeutic interventions.

## Appendix

Multimedia Appendix 1 Study clinics and investigators.

Multimedia Appendix 2 Change from baseline in the mean number of consultations in the exercise and conventional groups.

Multimedia Appendix 3 CONSORT-EHEALTH checklist (V 1.6.2).

## Abbreviations

CLBP: chronic low back pain
EQ-5D-5L: EuroQoL 5 Dimensions 5 Level
LBP: low back pain
MCID: minimal clinically significant difference
NRS: Numerical Rating Scale
PRO: patient-reported outcome
QoL: quality of life
QQ method: Quantity and Quality method
RDQ-24: Roland-Morris Disability Questionnaire
SPIRIT: Standard Protocol Items: Recommendations for Interventional Trials
TSK-11: Tampa Scale for Kinesiophobia
WPAI-GH: Work Productivity and Activity Impairment Questionnaire: General Health
