# Supplementary material for: Evaluation of the Effect of Patient Education and Strengthening Exercise Therapy Using a Mobile Messaging App on Work Productivity in Japanese Patients With Chronic Low Back Pain: Open-Label, Randomized, Parallel-Group Trial
Source: JMIR Mhealth Uhealth. 2022 May 16;10(5):e35867. doi: 10.2196/35867 (PMC9152720; doi:10.2196/35867)
Supplement: Multimedia Appendix 1 [file mhealth_v10i5e35867_app1.docx]

Table S1. Study clinics and investigators.

| No. | Study center | Department | Investigator |
| --- | --- | --- | --- |
| 1 | Taro Orthopedic Clinic | Orthopedics | Taro Kawakami |
| 2 | HIRO Ortho Clinic | Orthopedics | Hiroshi Katsuno |
| 3 | Ohsato Orthopedic Rehabilitation Clinic | Orthopedics,  Rehabilitation | Yuji Ohsato |
| 4 | Kawawaki Clinic | Primary care | Nobuhiko Kawawaki |
| 5 | Yoshino Clinic | Primary care | Masanori Yoshino |
| 6 | Yamaguchi Clinic | Orthopedics | Shinichi Yamaguchi |
| 7 | Miura Clinic | Primary care | Hirokazu Miura |
| 8 | Shibahara Orthopedics Sports Joint Clinic | Orthopedics | Motoi Shibahara |
| 9 | Tokiwa Clinic | Primary care | Takahiro Arai |
| 10 | KM Pain Clinic | Pain Clinic | Keiko Miyahara |
| 11 | Uemura Pain Clinic | Pain Clinic | Yuhei Uemura |
| 12 | Tsukahara Pain Clinic | Pain Clinic | Yoshiko Tsukahara |
| 13 | Sunto Orthopedics Clinic | Orthopedics | Manabu Shimizu |
| 14 | Komuro Orthopedics Clinic | Orthopedics | Hajime Komuro |
| 15 | Sunomata Clinic | Primary care | Toshiki Kumazawa |
| 16 | Nakagawa Orthopedics Clinic | Orthopedics | Tanefumi Nakagawa |
