# Supplementary figures and images for: Evaluation of the Effect of Patient Education and Strengthening Exercise Therapy Using a Mobile Messaging App on Work Productivity in Japanese Patients With Chronic Low Back Pain: Open-Label, Randomized, Parallel-Group Trial
Source: JMIR Mhealth Uhealth. 2022 May 16;10(5):e35867. doi: 10.2196/35867 (PMC9152720; doi:10.2196/35867)

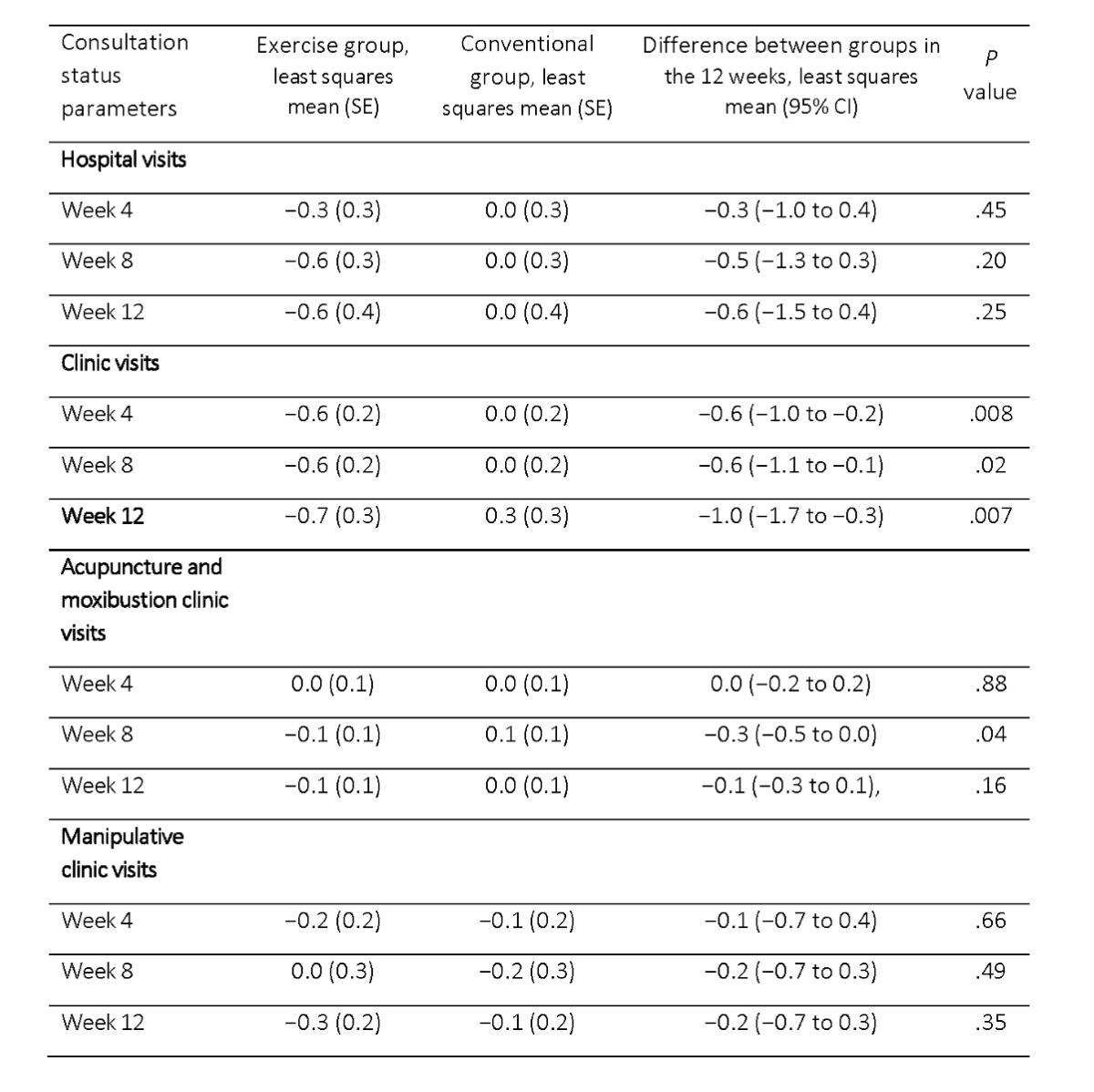

Supplement: Multimedia Appendix 2 [file mhealth_v10i5e35867_app2.png]
