# Supplementary material for: Evaluation of the Effect of Patient Education and Strengthening Exercise Therapy Using a Mobile Messaging App on Work Productivity in Japanese Patients With Chronic Low Back Pain: Open-Label, Randomized, Parallel-Group Trial
Source: JMIR Mhealth Uhealth. 2022 May 16;10(5):e35867. doi: 10.2196/35867 (PMC9152720; doi:10.2196/35867)
Supplement: Multimedia Appendix 3 [file mhealth_v10i5e35867_app3.pdf]

CONSORT-EHEALTH Checklist V1.6.2 Report	Manuscript Number	35867
**(based on CONSORT-EHEALTH V1.6), available at [http://tinyurl.com/consort-ehealth-v1-6].**		
**Date completed**		
3/17/2022 5:12:19		
**by**		
Naohiro Itoh		
Evaluation of the Effect of Patient Education and Strengthening Exercise Therapy Using Mobile Messaging Application on Work Productivity in Japanese Patients with Chronic Low Back Pain: Open-Label, Randomized, Parallel-Group Trial		
TITLE		
**1a-i) Identify the mode of delivery in the title**		
Yes. We described it in the title, “Using Mobile Messaging Application”.		
**1a-ii) Non-web-based components or important co-interventions in title**		
No. We did not mention it in the Title as we did not use non-web-based components.		
**1a-iii) Primary condition or target group in the title**		
Yes. We described it in the Title, “in Japanese Patients with Chronic Low Back Pain”.		
ABSTRACT		
**1b-i) Key features/functionalities/components of the intervention and comparator in the METHODS section of the ABSTRACT**		
No. We did not mention it in the Methods of the Abstract. However, we described it in the Objective of the Abstract: “ to explore the effects of online video patient education and strengthening exercise therapy using a mobile messaging application (app) on work productivity and pain in patients with chronic low back pain (CLBP) receiving pharmacological treatment".		
**1b-ii) Level of human involvement in the METHODS section of the ABSTRACT**		
No. We did not mention it in the Methods of the Abstract.		
**1b-iii) Open vs. closed, web-based (self-assessment) vs. face-to-face assessments in the METHODS section of the ABSTRACT**		
Yes. We described it in the Methods of the Abstract: “Patients with CLBP were randomly allocated to either the exercise group, who received education and exercise therapy using a mobile messaging app, or the conventional group.”, and “The endpoints were changes in work productivity, pain intensity, quality of life (QoL), fear of movement, and depression. The observation period for this study was 12 weeks.”.		
**1b-iv) RESULTS section in abstract must contain use data**		
Yes. We described it in the Results of the Abstract: “The exercise and conventional groups included 48 and 51 patients with a mean age of 47.9 years (27 patients, 56.3% male) and 46.9 years (28 patients ,54.9% male) in the full analysis set, respectively. No significant impact of these interventions on work productivity was observed in the exercise group compared to the conventional group (primary endpoint: Quantity and Quality method; 0.062 vs. 0.114; difference between groups: −0.053; 95% confidence interval (CI); −0.184 to-0.079; P=.43). However, the exercise group showed consistently better trends for other endpoints than the conventional group. Compared to the conventional group, the exercise group showed a significant improvement in the symptoms of low back pain (LBP) (3.2 vs. 3.8; difference between groups: -0.5; 95% CI: -1.1 to 0.0; P=.04), QoL (EQ-5D-5L: 0.068 vs. 0.006; difference between groups: 0.061; 95% CI: 0.008 to 0.114; P=.03), and fear of movement at Week 12 (-2.3 vs.0.5; difference between groups: -2.8; 95% CI: -5.5 to -0.1; P=.04).”.		
**1b-v) CONCLUSIONS/DISCUSSION in abstract for negative trials**		
Yes. We described it in the Conclusions: “This study does not reveal the effect of therapeutic interventions on CLBP on work productivity. Thus, further research is needed to assess work productivity with therapeutic interventions.”.		
INTRODUCTION		
**2a-i) Problem and the type of system/solution**		

Yes. We described it in the Introduction:” Despite these encouraging results, patients often show noncompliance with exercise therapy. Perceptions of the underlying illness and exercise therapy, lack of positive feedback, and the degree of helplessness are the factors related to noncompliance with exercise therapy [19]. In recent years, digital devices have become popular to support exercise therapy in musculoskeletal pain [20-22]. It has been reported that these digital devices improve adherence [23,24]. Most studies support the role of digital interventions for LBP alleviation [24-27].<br>The mobile messaging application (app) “Secaide” (Travoss Co., Ltd. (Tokyo, Japan)) is a digital device that is designed to enhance the patient’s understanding of CLBP and enable remote exercise therapy for more accessible and personalized home-based pain management. The app was nicknamed "se / ca / ide" by the "self-care guide service". "Secaide" also means “in the world” when read in Japanese. The usefulness of mobile messaging app-based interventions in managing neck/shoulder stiffness and LBP is established in workers in randomized controlled trials [28].”		
**2a-ii) Scientific background, rationale: What is known about the (type of) system**		
Yes. We described it in the Introduction:” Several studies have reported that exercise alleviates CLBP and disability [10-12]. Furthermore, it has been reported that exercise regimens reduce disability [13] and improve the QoL of individuals with CLBP [14,15]. Patients with chronic pain, including CLBP, exhibit various symptoms and signs as the duration of pain increases. When the pain lingers, it becomes intractable and serious through a cyclical interaction with psychosocial factors. As the fear-avoidance model of pain illustrates, pain often involves catastrophizing when it becomes intractable [16]. There are also several psychological treatments or therapies for musculoskeletal symptoms [17]. In a study on patients with CLBP, both groups—one that received only exercise therapy and the other that received a combination of cognitive-behavioral therapy and exercise therapy—showed improvements in pain intensity and QoL compared to baseline [18].”.		
**Does your paper address CONSORT subitem 2b?**		
Yes. We described it in the Introduction:” Previous studies have not clarified the impact of intervention in CLBP treatment on presenteeism in patients. As a hypothesis, we expected that therapeutic intervention for CLBP would have a positive effect on presenteeism. This study aimed to explore the effects of patient education and strengthening exercise therapy on work productivity, symptoms, and QoL in patients with CLBP who were receiving medication and continued to have pain despite treatment. As a new attempt, we used online videos for patient education and a mobile messaging app to support the continuation of exercise therapy. Under the influence of Coronavirus Disease 2019 (COVID-19), we devised methods for study continuation without any visits to clinics by the intervention in online remote exercise therapy and by using patient-reported outcomes (PROs) as an outcome evaluation method.”.		
METHODS		
**3a) CONSORT: Description of trial design (such as parallel, factorial) including allocation ratio**		
Yes. We described it in the Study Design: “This is a multicenter, open-label, randomized, parallel-group study conducted in Japan from June 2020 to March 2021 at 16 clinics (Supplement Table 1). The main clinical specialty of the 16 community-based clinics included 8 orthopedic facilities, 3 pain clinics, and 5 primary care facilities. In this study, patients were followed up for 12 weeks (Figure 1). Patients who met the selection criteria were randomly assigned using a stochastic minimization procedure with allocation regulators as age (under 45/45 years or older), sex (male/female), and willingness to enhance exercise therapy (yes/no).”.		
**3b) CONSORT: Important changes to methods after trial commencement (such as eligibility criteria), with reasons**		
We did not change the methods after the trial started.		
**3b-i) Bug fixes, Downtimes, Content Changes**		
We have not experienced any bug fixes, downtimes, and content changes.		
**4a) CONSORT: Eligibility criteria for participants**		
Yes. We described it in the Study Population: “Patients who met the following criteria were included in the study: (1) having LBP more than 3 months, (2) aged 20–64, (3) receiving prescribed pharmacological treatment for the pain, (4) not likely to experience any unexpected pain flare-ups for 12 weeks, (5) able to walk independently, (6) engaging in work for more than 3 days/week in either full time or part time capacity for more than 3 hours a day, and (7) having the skill and understanding to operate mobile communications. The CLBP diagnosis was established by qualified practicing physicians.<br>The key exclusion criteria were as follows: (1) aged >65 years, (2) having CLBP unrelated to a musculoskeletal condition, (3) with radiculopathy or constructive spinal deformity, (4) having LBP with red flags (with chest pain, malignant tumor, HIV infection, malnutrition, significant weight loss of 5% or more within 1 month, extensive neurological symptoms, or fever of 37.5℃ or higher)”.		
**4a-i) Computer / Internet literacy**		

We did not clarify the computer/Internet literacy in this study, because the participants were all patients who used their own smartphone in a daily basis. We just have included in the inclusion criteria that “having the skill and understanding to operate mobile communications.”		
**4a-ii) Open vs. closed, web-based vs. face-to-face assessments:**		
Yes. We described it in the Study Design: “This is a multicenter, open-label, randomized, parallel-group study conducted in Japan from June 2020 to March 2021 at 16 clinics (Supplement Table 1). The main clinical specialty of the 16 community-based clinics included 8 orthopedic facilities, 3 pain clinics, and 5 primary care facilities. In this study, patients were followed up for 12 weeks (Figure 1). Patients who met the selection criteria were randomly assigned using a stochastic minimization procedure with allocation regulators as age (under 45/45 years or older), sex (male/female), and willingness to enhance exercise therapy (yes/no).”.		
**4a-iii) Information giving during recruitment**		
We described it in the Study Design: “Written informed consent was obtained from all patients before enrollment in the study.”.		
**4b) CONSORT: Settings and locations where the data were collected**		
Yes. We described it in the Survey: “All patients were required to respond to an online survey that captured demographic and background information, including occupation and exercise habits. Furthermore, pharmacological and surgical treatment for CLBP and the number of institutional visits in the last 30 days were collected in Weeks 0–4, 4–8, and 8–12 and at study discontinuation.”		
**4b-i) Report if outcomes were (self-)assessed through online questionnaires**		
Yes. We described it in the Survey: “All patients were required to respond to an online survey that captured demographic and background information, including occupation and exercise habits. Furthermore, pharmacological and surgical treatment for CLBP and the number of institutional visits in the last 30 days were collected in Weeks 0–4, 4–8, and 8–12 and at study discontinuation.<br>Adherence to the use of mobile messaging app-based exercise therapy was measured by the rate of implementation (%) calculated as follows: (access days/observation period) x 100. Category aggregation for the adherence rate was done by 0%–25%, 25%–50%, 50%–75%, and ≥75%. Assessments were made from the log information (date) of Secaide and the PRO response date, that is, Weeks 0–4, 4–8, 8–12, and 0–12.”.		
**4b-ii) Report how institutional affiliations are displayed**		
No. It is not described in the manuscript.		
**5) CONSORT: Describe the interventions for each group with sufficient details to allow replication, including how and when they were actually administered**		
**5-i) Mention names, credential, affiliations of the developers, sponsors, and owners**		
Yes. We mentioned in the Introduction, Patient Education and Exercise Therapy of the Methods, and in the Conflicts of Interest:” The mobile messaging application (app) “Secaide” (Travoss Co., Ltd. (Tokyo, Japan)) is a digital device that is designed to enhance the patient’s understanding of CLBP and enable remote exercise therapy for more accessible and personalized home-based pain management.”, ” An online video program was used to provide evidence-based thinking about the importance of a cognitive-behavioral approach for CLBP with patients. The exercise therapy was developed by Travoss Co.," and "operation of intrinsic pain" for the "activation of endogenous substances by aerobic exercise" [30,31].<br>“Secaide,” a mobile messaging app for mobile communication devices such as smartphones and tablets, with download enabled by a QR code, is an aid to exercise therapy.” and “KM is a shareholder/an adviser of Trunk Solution Co., Ltd.”		
**5-ii) Describe the history/development process**		
Yes. We mentioned in the Introduction: “The mobile messaging application (app) “Secaide” (Travoss Co., Ltd. (Tokyo, Japan)) is a digital device that is designed to enhance the patient’s understanding of CLBP and enable remote exercise therapy for more accessible and personalized home-based pain management. The app was nicknamed "se · ca · ide" by the "self-care guide service". "Secaide" also means “in the world” when read in Japanese. The usefulness of mobile messaging app-based interventions in managing neck/shoulder stiffness and LBP is established in workers in randomized controlled trials [28].”.		
**5-iii) Revisions and updating**		
The system we used in this study were not revised and updated during the intervention.		
**5-iv) Quality assurance methods**		
We did not adopt and grasp quality assurance methods for the AI-assisted health program.		
**5-v) Ensure replicability by publishing the source code, and/or providing screenshots/screen-capture video, and/or providing flowcharts of the algorithms used**		

We did not adopt and grasp quality assurance methods for the AI-assisted health program.		
**5-vi) Digital preservation**		
In the Reference list of the manuscript, we showed the website of the AI-assisted health program, https://www.secaide.me/. In case the website disappears, an archived page is https://webcache.googleusercontent.com/search?q=cache:QTdPBshGJWQJ:https://www.secaide.me/+&cd=1&hl=ja&ct=clnk&gl=us. If you apply to use the AI-assisted health program, you can get access to the program because it is commercial.".		
**5-vii) Access**		
We described it in the Patient Education and Exercise Therapy of the Methods: "Secaide," a mobile messaging app for mobile communication devices such as smartphones and tablets, with download enabled by a QR code, is an aid to exercise therapy." If you apply to use the AI-assisted health program, you can get access to the program because it is commercial. Users were normally charged the fee. In this study, they were not charged. We also provided the reviewers introduction videos of the AI-assisted health system on secaide.".		
**5-viii) Mode of delivery, features/functionalities/components of the intervention and comparator, and the theoretical framework**		
We described it in the Patient Education and Exercise Therapy of the Methods: ""Secaide," a mobile messaging app for mobile communication devices such as smartphones and tablets, with download enabled by a QR code, is an aid to exercise therapy. In Japan, this mobile messaging app is used for text messages and voice calls [28]. Patient education and exercise therapy announcements were conducted as follows. The artificial intelligence-assisted chatbot was programmed to send messages to users with exercise instructions and some tips on what they can do in their daily lives to improve their symptoms. The messages were sent every day at a fixed time through the LINE app (a smartphone app widely used for sending and receiving text messages and making voice calls in Japan) (LINE Corporation, Tokyo, Japan). The notification time could be changed by the users to a time convenient for them. The exercise was performed at the patient's favorite time. The participants could finish their exercise within about 1–3 minutes each day (Supplement Figure 1-3).".		
**5-ix) Describe use parameters**		
We did not describe it in the manuscript, but previous studies have mentioned human involvement in Secaide. Please refer to reference 28.		
**5-x) Clarify the level of human involvement**		
We did not describe it in the manuscript, but previous studies have mentioned human involvement in Secaide. Please refer to reference 28.		
**5-xi) Report any prompts/reminders used**		
Yes. Although we didn't send specific reminders in addition to the program, we considered the messages that were sent from the chatbot every day at a fixed time also had reminder function.		
**5-xii) Describe any co-interventions (incl. training/support)**		
We described it in the Patient Education and Exercise Therapy of the Methods: "At each clinic, the conventional group received only routine medical care. In the exercise therapy group, in addition to the routine medical care, patient education and strengthening of exercise were provided.".		
**6a) CONSORT: Completely defined pre-specified primary and secondary outcome measures, including how and when they were assessed**		
We described it in the Primary Endpoint and Secondary Endpoints of the Study Endpoints in the Methods: "The primary endpoint was the change in work productivity at Week 12. The work productivity was measured using the Quantity and Quality method (QQ method) that evaluates work productivity in terms of quality, quantity, and efficiency and is an evaluation index for absenteeism [32]." and "The secondary endpoints were changes in work productivity measured using Work Productivity and Activity Impairment Questionnaire: General Health (WPAI: GH) [33], CLBP and shoulder stiffness (Numerical Rating Scale (NRS)) [34], subjective ratings of stiffness and LBP on a scale of 1 to 5 [28], disease-specific QoL (Roland-Morris Disability Questionnaire (RDQ-24)) [35,36], health-related QoL (EuroQoL 5 dimensions 5-level (EQ-5D-5L)) [3738], fear of movement (Tampa Scale for Kinesiophobia (TSK-11)) [39,40], degree of depression (Kessler Screening Scale for Psychological Distress (K-6)) [41], drug usage, and consultation status at medical institutions. All the secondary endpoints were measured at baseline and Week 12. Additionally, changes in LBP and drug usage were measured at Weeks 4 and 8 during the study period.".		
**6a-i) Online questionnaires: describe if they were validated for online use and apply CHERRIES items to describe how the questionnaires were designed/deployed**		
We did not validate the questionnaires for online use and apply CHERRIES items.		
**6a-ii) Describe whether and how "use" (including intensity of use/dosage) was defined/measured/monitored**		

We described it in the Survey of the Methods: “Adherence to the use of mobile messaging app-based exercise therapy was measured by the rate of implementation (%) calculated as follows: (access days/observation period) x 100. Category aggregation for the adherence rate was done by 0%–25%, 25%–50%, 50%–75%, and ≥75%. Assessments were made from the log information (date) of Secaide and the PRO response date, that is, Weeks 0–4, 4–8, 8–12, and 0–12.”.		
**6a-iii) Describe whether, how, and when qualitative feedback from participants was obtained**		
The owner of the program, Travoss, collected feedbacks from the participants of the program. If they had technical problems, they could ask the inquiry service of the program.		
**6b) CONSORT: Any changes to trial outcomes after the trial commenced, with reasons**		
Yes. We described it in the Survey: “All patients were required to respond to an online survey that captured demographic and background information, including occupation and exercise habits. Furthermore, pharmacological and surgical treatment for CLBP and the number of institutional visits in the last 30 days were collected in Weeks 0–4, 4–8, and 8–12 and at study discontinuation.”		
**7a) CONSORT: How sample size was determined**		
**7a-i) Describe whether and how expected attrition was taken into account when calculating the sample size**		
**7b) CONSORT: When applicable, explanation of any interim analyses and stopping guidelines**		
We described it in the Primary Endpoint and Secondary Endpoints of the Study Endpoints in the Methods: “The primary endpoint was the change in work productivity at Week 12. The work productivity was measured using the Quantity and Quality method (QQ method) that evaluates work productivity in terms of quality, quantity, and efficiency and is an evaluation index for absenteeism [32].” and “The secondary endpoints were changes in work productivity measured using Work Productivity and Activity Impairment Questionnaire: General Health (WPAI: GH) [33], CLBP and shoulder stiffness (Numerical Rating Scale (NRS)) [34], subjective ratings of stiffness and LBP on a scale of 1 to 5 [28], disease-specific QoL (Roland-Morris Disability Questionnaire (RDQ-24)) [35,36], health-related QoL (EuroQoL 5 dimensions 5-level (EQ-5D-5L)) [3738], fear of movement (Tampa Scale for Kinesiophobia (TSK-11)) [39,40], degree of depression (Kessler Screening Scale for Psychological Distress (K-6)) [41], drug usage, and consultation status at medical institutions. All the secondary endpoints were measured at baseline and Week 12. Additionally, changes in LBP and drug usage were measured at Weeks 4 and 8 during the study period.”.		
**8a) CONSORT: Method used to generate the random allocation sequence**		
We described it in the Study Design of the Methods: “This is a multicenter, open-label, randomized, parallel-group study conducted in Japan from June 2020 to March 2021 at 16 clinics (Supplement Table 1). The main clinical specialty of the 16 community-based clinics included 8 orthopedic facilities, 3 pain clinics, and 5 primary care facilities. In this study, patients were followed up for 12 weeks (Figure 1). Patients who met the eligibility criteria were randomly assigned using a stochastic minimization procedure with allocation regulators as age (under 45/45 years or older), sex (male/female), and willingness to enhance exercise therapy (yes/no).”.		
**8b) CONSORT: Type of randomisation; details of any restriction (such as blocking and block size)**		
We described it in the Study Design of the Methods: “This is a multicenter, open-label, randomized, parallel-group study conducted in Japan from June 2020 to March 2021 at 16 clinics (Supplement Table 1). The main clinical specialty of the 16 community-based clinics included 8 orthopedic facilities, 3 pain clinics, and 5 primary care facilities. In this study, patients were followed up for 12 weeks (Figure 1). Patients who met the eligibility criteria were randomly assigned using a stochastic minimization procedure with allocation regulators as age (under 45/45 years or older), sex (male/female), and willingness to enhance exercise therapy (yes/no).”.		
**9) CONSORT: Mechanism used to implement the random allocation sequence (such as sequentially numbered containers), describing any steps taken to conceal the sequence until interventions were assigned**		
We described it in the Study Design of the Methods: “This is a multicenter, open-label, randomized, parallel-group study conducted in Japan from June 2020 to March 2021 at 16 clinics (Supplement Table 1). The main clinical specialty of the 16 community-based clinics included 8 orthopedic facilities, 3 pain clinics, and 5 primary care facilities. In this study, patients were followed up for 12 weeks (Figure 1). Patients who met the eligibility criteria were randomly assigned using a stochastic minimization procedure with allocation regulators as age (under 45/45 years or older), sex (male/female), and willingness to enhance exercise therapy (yes/no).”.		
**10) CONSORT: Who generated the random allocation sequence, who enrolled participants, and who assigned participants to interventions**		

One of the authors, Dr. Yuki Yoshida generated the random allocation sequence. We also described it in the Study Design of the Methods and the Results: “This is a multicenter, open-label, randomized, parallel-group study conducted in Japan from June 2020 to March 2021 at 16 clinics (Supplement Table 1). The main clinical specialty of the 16 community-based clinics included 8 orthopedic facilities, 3 pain clinics, and 5 primary care facilities.” and “A total of 101 patients with CLBP were recruited, and consenting participants were randomly allocated to either the exercise group (50 randomized, 48 analyzed for efficacy) who used the online videos and Secaide for exercise therapy or the conventional group (51 randomized and analyzed) (Figure 2).”.		
**11a) CONSORT: Blinding - If done, who was blinded after assignment to interventions (for example, participants, care providers, those assessing outcomes) and how**		
**11a-i) Specify who was blinded, and who wasn’t**		
We described it in the Study Design of the Methods: “This is a multicenter, open-label, randomized, parallel-group study conducted in Japan from June 2020 to March 2021 at 16 clinics (Supplement Table 1). “.		
**11a-ii) Discuss e.g., whether participants knew which intervention was the “intervention of interest” and which one was the “comparator”**		
We described it in the Study Design of the Methods: “Patients who met the eligibility criteria were randomly assigned using a stochastic minimization procedure with allocation regulators as age (under 45/45 years or older), sex (male/female), and willingness to enhance exercise therapy (yes/no).”.		
**11b) CONSORT: If relevant, description of the similarity of interventions**		
We described it in the Patient Education and Exercise Therapy of the Methods: “At each clinic, the conventional group received only routine medical care. In the exercise therapy group, in addition to the routine medical care, patient education and strengthening of exercise were provided.”.		
**12a) CONSORT: Statistical methods used to compare groups for primary and secondary outcomes**		
Yes. We described it in the Statistical Analysis of the Methods: “Data were summarized using descriptive statistics of the mean (standard error (SE)) for continuous variables and frequencies and percentages for categorical variables. To compare continuous data in the two groups, an analysis of covariance (ANCOVA) model (covariates: treatment, baseline, age, sex, and willingness to exercise therapy) or mixed-effects model for repeated measures (MMRM) (covariates: treatment, baseline, time, time × treatment, age, sex, and willingness to exercise therapy) was used for the primary and secondary endpoints, depending on the times of measurements. Fisher’s exact test was used to compare percentages in the two groups.<br>In patients who had data reported at Week 12, post hoc analyses were performed to check the impact of the treatment compliance (<75%, ≥75%, and conventional group) on the primary endpoint (work productivity) and secondary endpoints (NRS of CLBP; RDQ-24). Data were analyzed using statistical analysis software (SAS) version 9.4 (SAS Institute Inc., Cary, NC).”.		
**12a-i) Imputation techniques to deal with attrition / missing values**		
We analyzed the data with 48 participants in the intervention group and 51 in the control. We had recruited 50 for intervention, but we lost 3 participants, before starting the intervention and we did not collect baseline information except for eligibility. There was no dropout in the control group. Therefore, we were obliged to perform the study for only 48 participants in the intervention group and 51 in the control (figure 2).		
**12b) CONSORT: Methods for additional analyses, such as subgroup analyses and adjusted analyses**		
Yes. We described it in the Statistical Analysis of the Methods: “In patients who had data reported at Week 12, post hoc analyses were performed to check the impact of the treatment compliance (<75%, ≥75%, and conventional group) on the primary endpoint (work productivity) and secondary endpoints (NRS of CLBP; RDQ-24). Data were analyzed using statistical analysis software (SAS) version 9.4 (SAS Institute Inc., Cary, NC).”.		
RESULTS		
**13a) CONSORT: For each group, the numbers of participants who were randomly assigned, received intended treatment, and were analysed for the primary outcome**		
Yes. We described it in the Study Population of the Results: “A total of 101 patients with CLBP were recruited, and consenting participants were randomly allocated to either the exercise group (50 randomized, 48 analyzed for efficacy) who used the online videos and Secaide for exercise therapy or the conventional group (51 randomized and analyzed) (Figure 2). Both groups continued with the prescribed pharmacological treatments.”.		
**13b) CONSORT: For each group, losses and exclusions after randomisation, together with reasons**		
Yes. We described it in the Study Population of the Results: “A total of 101 patients with CLBP were recruited, and consenting participants were randomly allocated to either the exercise group (50 randomized, 48 analyzed for efficacy) who used the online videos and Secaide for exercise therapy or the conventional group (51 randomized and analyzed) (Figure 2). Both groups continued with the prescribed pharmacological treatments.”.		
**13b-i) Attrition diagram**		

We described it as the Study Population of the Results: “More than 85% of the patients in both groups requested exercise therapy (exercise group: 42 out of 48 patients, conventional group: 45 out of 51 patients), which was a group highly conscious of exercise. Of the participants in the exercise group (n = 48), 37, 31, and 32 participants were adherent to the use of mobile messaging app-based exercise therapy in Weeks 0–4, 4–8, and 8–12, respectively (Figure 3).”.		
**14a) CONSORT: Dates defining the periods of recruitment and follow-up**		
Yes. We described it in the Study Design of the Methods, “This is a multicenter, open-label, randomized, parallel-group study conducted in Japan from June 2020 to March 2021 at 16 clinics (Supplement Table 1). The main clinical specialty of the 16 community-based clinics included 8 orthopedic facilities, 3 pain clinics, and 5 primary care facilities. In this study, patients were followed up for 12 weeks (Figure 1).”.		
**14a-i) Indicate if critical “secular events” fell into the study period**		
Yes. We described it in the Discussion, “It is possible that drastic changes in the working environment under the COVID-19 epidemic affected the assessment of work productivity. During the research period, the Government of Japan began to recommend remote work as a national policy. In the evaluation of work productivity, the quantity and quality of work at the time of evaluation point were compared with those when there was no CLBP. The effect of changes in working style might be greater than the effect of exercise therapy on work productivity. A survey of workers in remote work before and under the COVID-19 pandemic conducted in Japan in 2020 also reported that full remote work of five days a week reduced work productivity [50]. Therefore, the difference in work productivity between the two groups due to exercise therapy may not have been observed. In fact, many secondary endpoints showed a significant improvement in exercise therapy. However, the work productivities did not show a significant improvement. The work productivity assessments may have been particularly susceptible to COVID-19 compared to outcomes such as pain intensity and QoL. ”.		
**14b) CONSORT: Why the trial ended or was stopped (early)**		
There was no interruption or discontinuance during the intervention.		
**15) CONSORT: A table showing baseline demographic and clinical characteristics for each group**		
Yes. We described it in the Table 1, Baseline characteristics of participants.		
**15-i) Report demographics associated with digital divide issues**		
Yes. We described it in the Table 1, Baseline characteristics of participants.		
**16a) CONSORT: For each group, number of participants (denominator) included in each analysis and whether the analysis was by original assigned groups**		
**16-i) Report multiple “denominators” and provide definitions**		
Yes. We described all denominators in the manuscript, the figures, and tables.		
**16-ii) Primary analysis should be intent-to-treat**		
We performed intention to treat analyses for 48 participants in the intervention group and 51 in the control that we could collect data of the baseline survey.		
**17a) CONSORT: For each primary and secondary outcome, results for each group, and the estimated effect size and its precision (such as 95% confidence interval)**		
We described outcomes with 95% confidence interval in the Results: " At Week 12, the mean change (SE) in work productivity (QQ method) in the exercise group (n = 37) and the conventional group (n = 32) was 0.062 (0.069) and 0.114 (0.069), respectively (−0.053; 95% CI): −0.184, 0.079; P=.43).“, “Percent overall work impairment due to health in the exercise group (n = 36) and the conventional group (n = 26) was −13.3 (SE: 6.8) and −4.7 (SE: 7.6), respectively (−8.6; 95% CI: −23.6, 6.5; P=.26).”, “At Week 12, although no statistically significant difference in the reduction of the NRS scores was observed between the exercise (−1.1, SE: 0.3) and conventional groups (−0.7, SE: 0.4) (P=.26), mean subjective improvement in symptoms of CLBP was significantly greater in the exercise group (3.2, SE: 0.2) than that in the conventional group (3.8, SE: 0.3) (−0.5; 95% CI: −1.1, 0.0; P=.04).”.		
**17a-i) Presentation of process outcomes such as metrics of use and intensity of use**		
We described it as the Study Population of the Results: “More than 85% of the patients in both groups requested exercise therapy (exercise group: 42 out of 48 patients, conventional group: 45 out of 51 patients), which was a group highly conscious of exercise. Of the participants in the exercise group (n = 48), 37, 31, and 32 participants were adherent to the use of mobile messaging app-based exercise therapy in Weeks 0–4, 4–8, and 8–12, respectively (Figure 3).”.		
**17b) CONSORT: For binary outcomes, presentation of both absolute and relative effect sizes is recommended**		
We did not use binary outcomes for a questionnaire.		

**18) CONSORT: Results of any other analyses performed, including subgroup analyses and adjusted analyses, distinguishing pre-specified from exploratory**		
We described it in the Post Hoc Analysis of the Results, "As a post hoc analysis, the effects of exercise therapy on work productivity (QQ method), pain intensity, and RDQ-24 were examined in the group with a high compliance rate of exercise (≥75%) and the other groups (<75% compliance). At Week 12, patients who showed a higher (≥75%) adherence to exercise regimen had a greater improvement in work productivity (QQ method), NRS scores, and RDQ-24 than those with <75% adherence or the conventional group (Table 4).".		
**18-i) Subgroup analysis of comparing only users**		
We described it in the Post Hoc Analysis of the Results, "As a post hoc analysis, the effects of exercise therapy on work productivity (QQ method), pain intensity, and RDQ-24 were examined in the group with a high compliance rate of exercise (≥75%) and the other groups (<75% compliance). At Week 12, patients who showed a higher (≥75%) adherence to exercise regimen had a greater improvement in work productivity (QQ method), NRS scores, and RDQ-24 than those with <75% adherence or the conventional group (Table 4).".		
**19) CONSORT: All important harms or unintended effects in each group**		
We did not have any or unintended effects.		
**19-i) Include privacy breaches, technical problems**		
We did not have privacy breaches, technical problems during the intervention.		
**19-ii) Include qualitative feedback from participants or observations from staff/researchers**		
We did not have specific problems during the innervation.		
DISCUSSION		
**20) CONSORT: Trial limitations, addressing sources of potential bias, imprecision, multiplicity of analyses**		
**20-i) Typical limitations in ehealth trials**		
We stated it in the Limitation: "This study has certain limitations. Changes in work quality and quantity were used as outcomes for work productivity. This study was conducted during the COVID-19 pandemic when the social working environment evolved with the adoption of remote working. Furthermore, these changes in the work environment may have influenced the evaluation of work productivity.".		
**21) CONSORT: Generalisability (external validity, applicability) of the trial findings**		
**21-i) Generalizability to other populations**		
We stated it in the Discussion: " Using digital devices, the enhancement of exercise therapy gave better results in more endpoints than in routine clinical practice. These results and compliance rates are due to research conditions. Although the impact of these on treatment cannot be evaluated correctly, it is hoped that they will give an opportunity to think about the usefulness of remote medical care in CLBP.".		
**21-ii) Discuss if there were elements in the RCT that would be different in a routine application setting**		
The Ai-assisted health program was fully automated and we did not provide any additional human support except for inquiries about changes in their physical condition and the technical problems during the intervention.		
**22) CONSORT: Interpretation consistent with results, balancing benefits and harms, and considering other relevant evidence**		
**22-i) Restate study questions and summarize the answers suggested by the data, starting with primary outcomes and process outcomes (use)**		
We described it in the Discussion: " The online video patient education and strengthening exercise therapy using the mobile messaging app did not show any significant changes in work productivity or loss of workdays due to CLBP at Week 12 compared to the conventional pharmacological treatment in this study. To the best of our knowledge, there is no randomized controlled trial with the intervention outcome to improve work productivity in patients with CLBP, so this result cannot be compared with previous studies.".		
**22-ii) Highlight unanswered new questions, suggest future research**		
We stated in the Conclusions: " This study does not reveal the effect of therapeutic interventions on CLBP on work productivity. Further research is needed to assess work productivity with therapeutic interventions.".		
Other information		
**23) CONSORT: Registration number and name of trial registry**		
Yes. We mentioned in the Study Design of the Methods: "The study was registered with the UMIN Clinical Trials Registry (UMIN000041037)."		
**24) CONSORT: Where the full trial protocol can be accessed, if available**		

Major protocol of this study were written on the manuscript.		
**25) CONSORT: Sources of funding and other support (such as supply of drugs), role of funders**		
Yes. We wrote it in the Funding information of the Acknowledgements: “This work was supported by Shionogi & Co., Ltd., Osaka, Japan." and "The authors thank Dr. Tarveen Jandoo and Raghuraj Puthige, PhD of Enago Life Sciences for medical writing and editorial support in the preparation of this manuscript, which was funded by Shionogi & Co., Ltd. ".		
**X26-i) Comment on ethics committee approval**		
Yes. We described it in the Study Design if the Methods, “The study protocol and all subsequent amendments were approved by the relevant institutional review boards or independent ethics committees. The study was registered with the UMIN Clinical Trials Registry (UMIN000041037).”.		
**x26-ii) Outline informed consent procedures**		
Yes. We described it in the Study Design if the Methods, “The study was conducted in accordance with all the international and local laws, the principles of the “Declaration of Helsinki,” and the Standard Protocol Items: Recommendations for Interventional Trials (SPIRIT) statement [29]. Written informed consent was obtained from all patients before enrollment in the study.”.		
**X26-iii) Safety and security procedures**		
We mentioned it in the informed consent document.		
**X27-i) State the relation of the study team towards the system being evaluated**		
